# Graphene Oxides Derivatives Prepared by an Electrochemical Approach: Correlation between Structure and Properties

**DOI:** 10.3390/nano10122532

**Published:** 2020-12-17

**Authors:** Carlos Sainz-Urruela, Soledad Vera-López, María Paz San Andrés, Ana M. Díez-Pascual

**Affiliations:** 1 Department of Analytical Chemistry, Physical Chemistry and Chemical Engineering, Faculty of Sciences, University of Alcalá, Alcalá de Henares, 28805 Madrid, Spain; carlos.sainz@uah.es (C.S.-U.); soledad.vera@uah.es (S.V.-L.); mpaz.sanandres@uah.es (M.P.S.); 2 Institute of Chemistry Research, “Andrés M. del Río” (IQAR), University of Alcalá, Ctra. Madrid-Barcelona Km. 33.6, Alcalá de Henares, 28805 Madrid, Spain

**Keywords:** graphene oxide, oxidation level, structure-property relationship, surface topography, specific surface area, interlayer spacing, thermal stability, mechanical properties

## Abstract

Graphene oxide (GO) can be defined as a single monolayer of graphite with oxygen-containing functionalities such as epoxides, alcohols, and carboxylic acids. It is an interesting alternative to graphene for many applications due to its exceptional properties and feasibility of functionalization. In this study, electrochemically exfoliated graphene oxides (EGOs) with different amounts of surface groups, hence level of oxidation, were prepared by an electrochemical two-stage approach using graphite as raw material. A complete characterization of the EGOs was carried out in order to correlate their surface topography, interlayer spacing, defect content, and specific surface area (SSA) with their electrical, thermal, and mechanical properties. It has been found that the SSA has a direct relationship with the d-spacing. The EGOs electrical resistance decreases with increasing SSA while rises with increasing the D/G band intensity ratio in the Raman spectra, hence the defect content. Their thermal stability under both nitrogen and dry air atmospheres depends on both their oxidation level and defect content. Their macroscopic mechanical properties, namely the Young’s modulus and tensile strength, are influenced by the defect content, while no correlation was found with their SSA or interlayer spacing. Young moduli values as high as 54 GPa have been measured, which corroborates that the developed method preserves the integrity of the graphene flakes. Understanding the structure-property relationships in these materials is useful for the design of modified GOs with controllable morphologies and properties for a wide range of applications in electrical/electronic devices.

## 1. Introduction

Lately, considerable interest has been directed to graphene (G) and its derivatives including graphene oxide (GO). This oxidized form of graphene with several surface oxygen-containing groups such as epoxides, alcohols, and carboxylic acids is currently being used in a wide number of applications such as batteries, electrical cells, nanocomposites, besides to gain importance in biomedicine [1,2,3,4,5,6]. The greatest interest in GO over G could be explained by its higher possibilities to achieve functional properties since it is feasible to tailor its functionalities through reduction and functionalization processes [7,8]. Further, it is possible to prepare novel thin films and flexible composites at a cheaper cost compared to other carbon-based nanomaterials [9,10]. These materials can be used as fillers in polymer matrices due to their 2D lamellar structure, very high specific surface area (SSA), and their nature to disperse in a wide range of organic and inorganic solvents [11,12,13,14].

The control of the oxidation level can be useful for the design of GOs with tailorable structural, electrical, mechanical, thermal, and optical properties. For instance, GO is typically electrical insulating, albeit by controlling the synthesis conditions, its conductivity can be improved and conducting or semiconducting products, such as composites and thin films, can be developed [15,16,17]. The experimental conditions set for the synthesis process are essential, since they modify a number of physical properties including interlayer distance, specific surface area (SSA), defect content, and so forth. Hence, from an application viewpoint, it is crucial to understand how the change in those conditions affects the properties of GO-based nanomaterials [18]. To the best of our knowledge, very few studies have focused on investigating the correlation between SSA, interlayer spacing, and defect content with the mechanical, physical, and electrical properties of such nanomaterials [19]. In particular, SSA can directly influence the electrical properties, hence graphene materials with a large SSA are highly desirable. On the other hand, the GO band gap rises as the C/O ratio diminishes [20], which offers an effective approach to tailor the optical properties of these nanomaterials. Besides, as the oxidation level increases, both the Young’s modulus and tensile strength are expected to drop gradually owed to the breakage of the sp^2^ carbon network and the reduction in the energetic stability for the ordered GO [21]. Nonetheless, the rise in the GO oxidation level is beneficial for enhancing the mechanical properties of nanocomposites [13], particularly for polymeric matrices with oxygenated groups such as chitosan [22] or polyamide [23]. The heat capacity, thermal conductivity, and specific capacitance can also be modified via tailoring the level of oxidation [24].

In general, any carbon source can be used for the oxidation to GO, such as graphite with ordered layers, or expanded graphite. The first GO synthesis is attributed to Brodie and [25] Staudenmaier, Hummers, and Offeman [26], who obtained graphite oxide via the oxidation of graphite through various techniques. The method reported by Brodie involves mixing of graphite with sodium chlorate followed by treatment with nitric acid. Nonetheless, this oxidation process is kinetically restricted owing to phase transfer processes [27]. Hummers and Offeman carried out modifications on the initial two techniques to make them safer, such as the use of KMnO_4_ as an oxidizer instead of KClO_3_, which evolves toxic gas, and the addition of NaNO_3_ to form HNO_3_ in situ instead of using this acid as a solvent. Therefore, the Hummers’ method is the most widely used owed to its safer and more scalable nature. Any method that makes changes to the synthesis pathway proposed by Hummers can be regarded as a “modified Hummers method” [28]. For instance, approaches without NaNO_3_ to prevent the development of toxic gasses (NO_2_/N_2_O_4_) and to make easier the disposal of wastewater owed to the absence of Na^+^ and NO_3_^−^ ions have been reported. Further, maintaining a low density of lattice defects in GO (ca. below 1%) involves kinetic control over the oxidation reaction, which was attained by different strategies such as decreasing the oxidation temperature below 10 °C [27]. Sonication, stirring, or even rapid freezing have also been used to obtain GO from an aqueous solution of graphite oxide.

Chemical oxidation approaches involve time-consuming procedures, require aggressive reagents, and carefully controlled operating temperatures, which lead to expensive GO when it is synthesized at an industrial level. Lately, electrochemical processes have arisen as simple, environmentally friendly, and versatile alternatives to synthesize carbon nanomaterials due to their efficiency and low cost, which can overcome the issues mentioned above [29]. Graphite oxidation takes place at the anode, while the reduction occurs at the cathode. Besides, anion intercalation occurs at the anode, and cation intercalation at the cathode. In general, the electrochemical oxidation proceeds similarly to the chemical oxidation of graphite: First intercalation takes place, followed by covalent bond formation (oxidation). Different electrochemical methods have been reported, including two-electrode, three-electrode, and electrolyte exfoliation, which is the most suitable for industrial purposes [30]. Previous works have produced GO via electrochemical oxidation of different graphitic materials including graphite powders, rods, foils, plates, or even pencil cores [31,32,33]. By controlling the synthesis conditions, such as voltage, current intensity, exfoliation time, electrolyte composition, and temperature, GOs with various levels of oxidation, defect content, SSA, and interlayer spacing can be obtained [34]. Nonetheless, the electrolysis procedure usually deteriorates the delamination of the graphitic materials, yielding products with poor exfoliation levels, hence properties that significantly differ from those of GOs prepared by the abovementioned traditional methods. Further, it is typically difficult to precisely control the structural and physical properties of the resulting graphene materials, resulting in low-yield production [35], especially with methods using three electrodes (i.e., graphite as the working electrode, platinum as the counter electrode, and saturated calomel electrode as the reference one). In addition, most of the electrolytes used to date to prepare GO imply some drawbacks, such as deficient oxidation, inadequate intercalation, and difficult removal, thus the electrolyte choice is decisive. The separation and purification of the electrochemical synthesized GOs from the as-produced product is another challenging task [27]. To synthesize GO with higher crystallinity and oxidation degree, photosynergetic electrochemical methods have been reported that used oxalate anions as both intercalation ions and co-reactant. This promoted the interfacial concentration of hydroxyl radicals generated, especially under illumination, and the oxidation degrees obtained were comparable to those of a GO prepared by the Hummers’ method [36], albeit increased the complexity of the method. Overall, notwithstanding remarkable research has focused on GO synthesis via electrochemical exfoliation from graphite, very few investigations have succeeded. Consequently, additional research in this direction is desired to develop straightforward, scalable, safe, fast, inexpensive, and environmentally friendly electrochemical methods. In particular, a reproducible process able to finely control the chemical composition and structure of the resulting GO nanomaterials is required.

In this regard, two main issues should be addressed. On the one hand, to the best of our knowledge, no facile electrochemical exfoliation procedure able to accurately control the structure and chemical composition of the synthesized EGOs has been developed so far. The published methods usually result in non-homogeneous mixtures comprising traces from the raw materials, layers with diverse defect content, lateral size, and number of oxygenated groups hence level of oxidation. On the other hand, the reported approaches also deal a compromise between the reaction yield and the property control of the graphene derivatives. Tuning up inexpensive electrochemical exfoliation approaches to synthesize GOs aimed at specific applications is still an open question.

In a previous study [37], we reported a novel, straightforward, green, and inexpensive two-stage approach for the synthesis of electrochemically exfoliated graphene oxides (EGOs) that allows to finely control the level of GO oxidation and exfoliation by carefully modifying the synthesis conditions. A broad range of experimental conditions were tested to optimize the synthesis route in order to attain homogeneous EGOs that preserved the integrity of the graphene flakes at a good yield. The EGOs preparation process comprised two steps: Firstly a mild intercalation stage of SO_4_^2−^ ions within the graphite sheets, resulting in a graphite intercalation compound (GIC) and then an oxidation/exfoliation stage of the GIC under stronger conditions. The electrolyte concentration, voltage, and time of both stages were modified to evaluate their influence on the defect content and level of oxidation of the resulting EGOs, and an unprecedented minimum C/O value was obtained for the optimal conditions. Further, results were compared to those obtained for a reference GO synthesized via a modified Hummers’ method.

The aim of the current work is to carry out a complete characterization in order to correlate the surface topography, interlayer spacing, defect content, and specific surface area (SSA) of the synthesized EGOs with their macroscopic electrical, thermal, and mechanical properties. Thus, the EGOs electrical resistance has been found to be dependent on their SSA and defect content, their thermal stability on both their oxidation level and defect content, and their Young’s modulus was influenced by their number of defects as well. However, no correlation was found between their mechanical properties and their SSA or interlayer spacing. Understanding the structure–property relationships in these materials is useful for the design of modified GOs with controllable morphologies and properties for a wide range of applications, particularly in electrical/electronic devices.

## 2. Materials and Methods

### 2.1. Materials and Reagents

Flexible graphite foil (FGF, d_25 °C_ = 1.00 g/cm^3^, C: 99.5%, S < 300 ppm, Cl < 50 ppm, ash < 1%, thickness 0.1 mm) was supplied by Beyond Materials, Inc. (Tucson, AZ, USA) and dried in an oven at 60 °C for 48 h before use. Powdered graphite flakes (SP-1, d_25 °C_ = 1.05 g/cm^3^, C: 99.9%, ash < 0.5%, average size 30–150 μm) were purchased from Bay Carbon, Inc. (Michigan, MI, USA) and dried under identical conditions. KMnO_4_, H_2_SO_4_, K_2_S_2_O_8_, P_2_O_5_, H_2_O_2_ (30 wt% in water), and platinum wire, (ø: 0.5 mm, 99.99% trace metals basis) were obtained from Sigma-Aldrich (Madrid, Spain) and used as received. Ultrapure water was purified by a Millipore Elix 15,824 Advantage 15 UV system (Millipore, Milford, CT, USA).

### 2.2. Preparation of GOs via a Two-Stage Electrochemical Process

Electrochemically exfoliated graphene oxides (EGOs) were prepared from FGF at room temperature (25 ± 2 °C) through an electrochemical procedure that included two stages: The first was performed in an electrolysis cell with a slice of FGF fixed onto a tungsten wire by silver glue as anode, a Pt wire as cathode, and 98 wt% H_2_SO_4_ diluted in 100 mL of Milli-Q water as an electrolyte. A voltage of 1 or 2 V was initially applied for 10 or 30 min, leading to formation of a graphite intercalation compound (GIC). The second stage consisted in the electrochemical oxidation of the GIC that acted as anode, a Pt wire as cathode, and 40, 65, or 98 wt% H_2_SO_4_ as an electrolyte. A high voltage in the range of 10 to 30 V was applied for periods between 30 and 120 s.

The synthesized EGOs were collected by filtration, washed with water, purified via centrifugation at 2500 rpm, and ultrasonicated for 30 min at a power of 140 W, leading to well-dispersed EGO. A more detailed description of the synthesis procedure is given in [37]. The nomenclature of the EGO samples and the experimental conditions for their synthesis are collected in Table 1. For comparative purposes, a reference GO was synthesized from graphite via a modified Hummers’ method, as reported elsewhere [32], using a mixture of concentrated K_2_S_2_O_8_, H_2_SO_4_, and P_2_O_5_. The product was filtered, dried, and oxidized again through addition of H_2_SO_4_, KMnO_4_ and cold water.

### 2.3. Characterization

A LECO CHNS-932 elemental analyzer was used to perform elemental analysis measurements.

The morphology was analyzed with a SU8000 Hitachi scanning electron microscope (Hitachi High-Technologies, Tokyo, Japan), operating at 15.0 kV and an emission current of 10 mA.

Atomic force microscopy (AFM) imaging was performed using a Bruker Dimension Icon system coupled with a Nanoscope V controller (Bruker, Karlsruhe, Germany), using Peakforce QNM imaging mode and a 100 μm long monolithic silicon cantilever.

X-ray diffraction (XRD) measurements were carried out on a Bruker D8 Advance diffractometer (Bruker, Karlsruhe, Germany), fitted with a Cu X-ray tube and a Ni K_β_ filter operating at 40 kV and an intensity of 40 mA.

Raman spectra were acquired at room temperature with a laser output power of 1 mW using a Renishaw Raman microscope (Renishaw plc., Gloucestershire, UK), incorporating a He-Ne laser (632.8 nm). To minimize the signal-to-noise ratio, at least 10 scans were recorded for each sample. Data were then processed with the WiRE 3.3 Renishaw software, and the spectra were normalized to the G band for the sake of comparison.

The thermal stability of the samples was measured with a TA Instruments Q50 thermobalance (TA Instruments, Barcelona, Spain), via thermogravimetric analysis (TGA) experiments under both oxidative (dry air) an inert (N_2_) atmosphere. Measurements were performed from 100 to 800 °C at a heating rate of 10 °C/min, with a gas flow rate of 60 mL/min. Prior to the tests, samples were dried for 72 h and then placed inside aluminum pans.

Brunauer–Emmett–Teller (BET) specific surface area analysis was performed via nitrogen adsorption–desorption measurements at 77 K with a Quantachrom Autosorb IQ-C (Anton Paar GmbH, Graz, Austria) gas adsorption system. Prior to the analysis, moisture content was removed by drying for 2 days at 80 °C.

The electrical resistivity of the synthesized EGOs was determined at room temperature under a pressure of 600 kPa set by using an upper weight, with a KEITHLEY 2182A nanovoltmeter and a KEITHLEY 6221 current source (Tektronix, Inc., Beaverton, OR, USA). Prior to the measurements, each sample was positioned in a Teflon cylinder and compressed for 1 h between two stainless-steel plates that acted as electrodes. R_s_ was calculated as: R_s_ = 4532 × (V/I), being V the test voltage and I the current.

A standard test method for measuring the tensile properties of thin films, ASTM D882, was used for testing the mechanical properties of the EGOs. Tensile testing was carried out with an Instron 5565 Testing Machine. A 1 kN load cell was used and the crosshead speed was 10 mm min^−1^. The results reported are the mean values for six replicates. Uniform thin films were prepared via solution-casting of aqueous EGOs suspensions onto chemically and ultrasonically cleaned glass substrates.

## 3. Results and Discussion

### 3.1. Topography of the Synthesized EGOs

**Figure 1:**
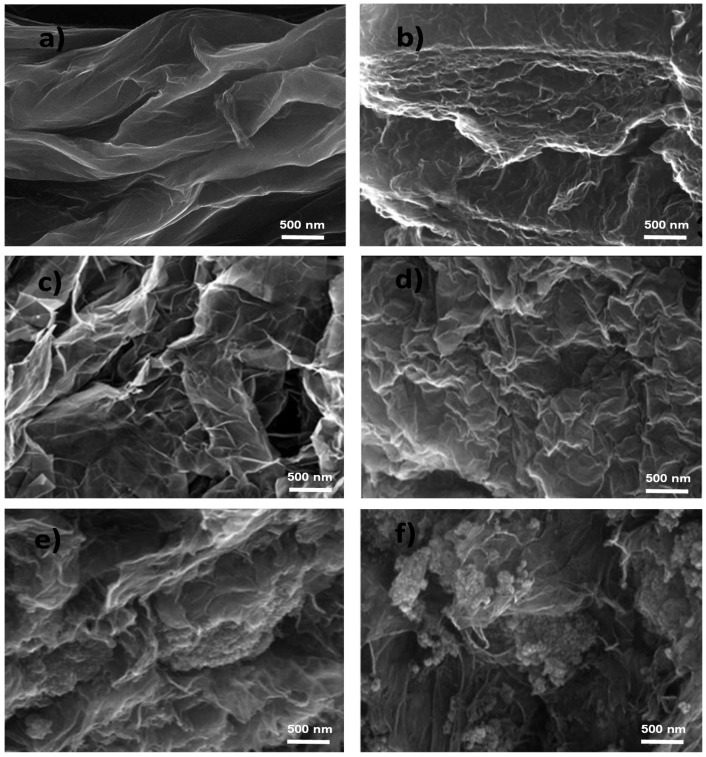
Representative SEM images of EGO 14 (**a**), EGO 15 (**b**), EGO 5 (**c**), reference GO * (**d**), EGO 2 (**e**), and EGO 21 (**f**).

The surface topography of FGF and the synthesized EGOs was examined by SEM, and typical images of the reference sample synthesized by the Hummers’ method (GO *), EGO 2, EGO 5, EGO 14, EGO 15 and EGO 21 are compared in Figure 1.

The image of EGO 14 (Figure 1a), obtained by applying a low bias of 2 V for 10 min followed by oxidation under 20 V for 60 s using 65% H_2_SO_4_ as electrolyte, reveals a rough surface topography with well-exfoliated and very well detached graphene sheets, with thicknesses in the range of 5–15 nm. Furthermore, the flakes appear wrinkled, indicating a deformation of the graphene layers due to the linkage of the oxygenated functional groups subsequent to the electrochemical process. In addition, the integrity of the graphene flakes is preserved, showing large area and homogeneous GO sheets. Analogous curvatures and surface folds have been previously reported for GO functionalized with amines [38], ascribed to increased number of regular hydrogen bonds between the amine moieties. Thus, EGO 14 presents a low C/O ratio (Table 1), that is, a high oxidation level, likely with a very high content of carboxylic acid groups [37], preferentially located at the sheet edges, which are able to interact via hydrogen bonding. Such surface wrinkling could be a crucial advantage for use in catalytic reactions, due to the higher accessibility of the catalytic sites.

A good exfoliation level is also found for EGO 15 (Figure 1b), in which the electrolyte concentration was increased to 98% and the rest of the conditions were maintained (Table 1), hence it exhibits higher oxidation level. Further, its average layer thickness was found to be slightly thicker than that of EGO 14, and the sheets appear less separated. On the other hand, a less efficient exfoliation and oxidation, albeit with considerably folding, is found for EGO 5 (Figure 1c), in which the low voltage was decreased to 1 V and the other conditions were similar to those of EGO 14. This indicates that the voltage of the first stage is critical to achieve a good wetting of the sample and to allow the intercalation of ions within the graphite sheets, thus promoting their exfoliation. A denser and more compact structure is also observed for the reference GO synthesized by the Hummer’s method (Figure 1d), which shows a C/O ratio of 2.25, and layer thicknesses up to 30 nm in good agreement with previous works [26].

On the other hand, the image of EGO 2 (Figure 1e) reveals a mild exfoliation, with connected flakes forming a compact network, likely related to the fact that the low voltage was applied for a long time. Thus, the FGF sheets could be damaged and broken into small pieces during the intercalation stage, thereby resulting in poorly exfoliated sheets, with a strongly restacked sheet arrangement. Regarding EGO 21 (Figure 1f), in which the voltage of the second stage is higher than 20 V, large GO aggregates can be observed, since the exfoliation rate should be very fast, also resulting in thick flakes (i.e., ≥20 nm) and a low SSA. This will be reflected in a decrease in mechanical strength and conductivity, as will be discussed in following sections. Likely, only the lateral parts of the sheets are oxidized, thus resulting in a very high C/O ratio (low oxidation degree).

**Figure 2:**
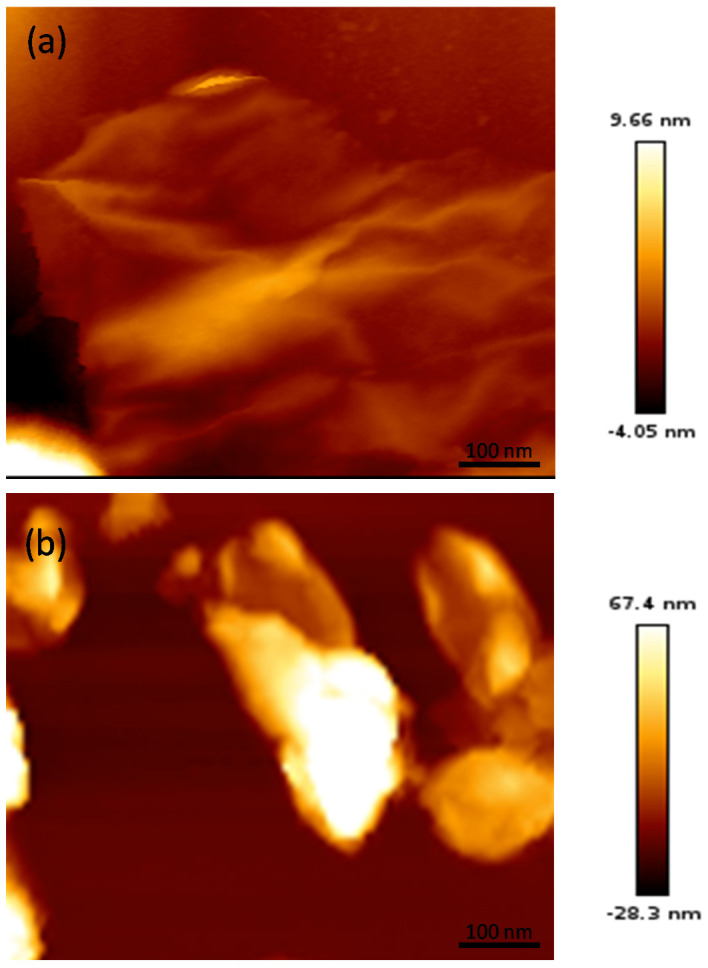
Representative atomic force microscopy (AFM) images of EGO 15 (**a**) and EGO 21 (**b**).

Further information about the surface topography of the EGOs was obtained from AFM, and representative images of EGO 14 and EGO 21 are compared in Figure 2.

The image of EGO 14 clearly reveals the formation of very thin, homogeneous, and good-quality GO sheets, without traces of the pristine material. Very well exfoliated and wrinkled sheets can be detected, with a high degree of bending, in agreement with the presence of many oxygenated functional groups that can interact via H-bonding [39]. Thus, the application of 20 V for 60 s using H_2_SO_4_ as electrolyte is proven to be a very efficient exfoliation method. Regarding EGO 15, prepared under the same conditions yet using concentrated H_2_SO_4_ as electrolyte, the sheets were found to be slightly thicker, albeit their exfoliation level was also very good, and the integrity of the graphene flakes was also preserved. Nonetheless, blocks of GO aggregates were detected for EGO 21, with flatter and smoother surfaces. The sheets could have broken during the second stage of the electrochemical process, resulting in sheets with smaller lateral sizes. Further, the presence of defects in the GO sheets can result in physical holes which generate poor waviness in the π-system [40]. Overall, both techniques point that the EGOs with the lowest C/O ratio, that is, the highest level of oxidation (i.e., EGO 15) display the best exfoliation and the lowest flake thickness.

### 3.2. Specific Surface Area of the EGOs

**Figure 3:**
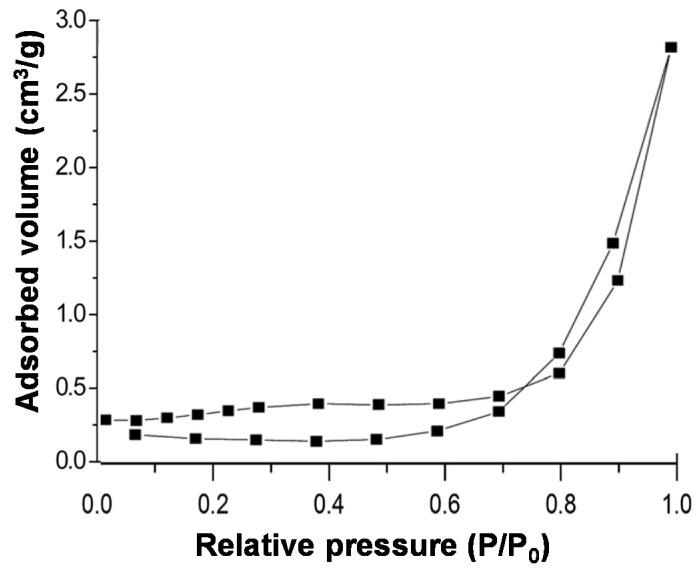
Nitrogen adsorption–desorption isotherm for EGO 21.

The specific surface area (SSA) for the reference GO and the synthesized EGOs was measured via the nitrogen adsorption–desorption technique. Figure 3 shows, as an example, the isotherm obtained for EGO 21. According to the International Union of Pure and Applied Chemistry (IUPAC) classification, the isotherm displays the characteristics of both type-III and type-V isotherms [41]. A similar curve shape was found for all the EGOs, which present SSA values in the range of 9 to 68 m^2^ g^−1^. The EGO 21 exhibits the lowest SSA, in agreement with the intense agglomeration of GO flakes as revealed by SEM and AFM. Conversely, EGO 14 displays the highest SSA, consistent with its improved exfoliation and well separated sheets. It has been reported that depending on the synthesis conditions such as temperature, electrolyte type and concentration, time, etc., SSA of GO can range from 4 up to 360 m^2^ g^−^^1^ [42]. For all the EGOs, the BET surface area was smaller than 70 m^2^ g^−1^, which could be expected for electrochemically synthesized GO materials that normally exhibit relatively low values due to a highly restacked sheet arrangement with a wrinkled texture.

**Figure 4:**
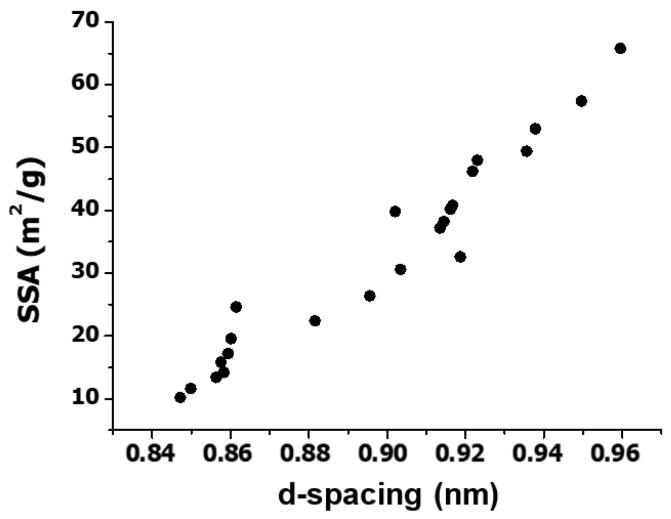
Specific surface area (SSA) vs. d-spacing for the synthesized EGOs.

In order to obtain more information about the parameters influencing SSA, this physical property was plotted against the interlayer distance (also called d-spacing) corresponding to the (001) reflection obtained from the X-Ray diffractograms (Table 1) [37], and the results are plotted in Figure 4. A very good correlation is found between these two properties: As the d-spacing increases, the SSA rises. Thus, the largest interlayer spacing, close to 0.96 nm, is obtained for EGO 14, which displays the highest SSA, while the smallest, about 0.85 nm, are found for EGO 19 and 21. On the other hand, EGO 4 and 5, prepared under mild conditions, present in between *d* and SSA values. This means that the d-spacing grows with increasing the available surface area of the EGOs, suggesting that their structure becomes more regular and orientated. An analogous trend of increase in SSA with the interlayer distance has been reported for thermally reduced GO synthesized in the presence of CTAB surfactant [43], which are intercalated between the nanomaterial layers. In our work, the intercalation of SO_4_^2−^ ions within the graphite layers during the first synthesis stage also results in significant interlayer spacing, and as it increases, the SSA becomes larger.

**Figure 5:**
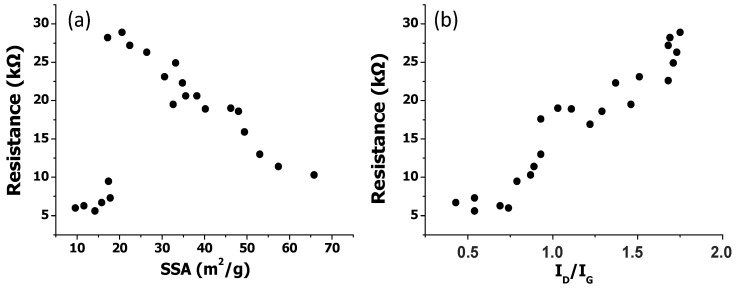
Electrical resistance vs. specific surface area SSA (**a**), and vs. I_D_/I_G_ band intensity ratio obtained from the Raman spectra (**b**).

To get insight about the influence of SSA on a bulk property, the electrical conductivity, the EGOs electrical resistance was measured and plotted against this parameter (Figure 5). In general, the electrical resistance decreases, that is, the conductivity increases with increasing SSA. This behavior is expected considering that the increased surface area of the stacked GO sheets implies better degree of exfoliation, leading to a greater chance of forming percolated networks, hence enhanced electron mobility. Improved electrical conductivity with increasing SSA has been previously reported for graphene-based porous materials synthesized by a green process [44]. This confirms that bulk properties are highly dependent on SSA, and consequently, on the sheet microstructure and also interlayer distance.

The mechanism of electron charge transport in graphene materials is not well understood yet. It has been reported that charge will percolate by a hopping conduction mechanism [45], which consists in the transport of charges via localized states. However, other mechanisms including the development of highly conductive assemblies/paths as well as ionic channels facilitating charge transfer through the sample have been proposed [46]. In our work, the presence of SO_4_^2−^ ions within the graphite layers could also promote charge transfer, accounting for the fact that increased interlayer distance, hence increased SSA imply better conductivity.

It should be noticed that an anomalous behavior is found for samples with very low SSA values (<20 nm), since they display very high electrical conductivity (Figure 5a). This could be rationalized considering that these EGOs present the highest C/O ratios (Table 1), that is, the lowest oxidation level, hence fewer sp^3^ carbon atoms and consequently higher aromaticity. To confirm this hypothesis, the electrical resistance was also plotted against the I_D_/I_G_ ratio (the integrated intensity ratio of the D peak and the G peak in the Raman spectrum, which is indicative of the quality of the GO layers and their defect content [47]). Thus, the G band arises from the E2g vibrational mode found in graphite single crystal, and it is characteristic of sp^2^ hybridization while the D band is related to defects, vacancies, or lattice disorders due to the binding of oxygenated groups.

A direct relationship can be found between the electrical resistance and the I_D_/I_G_ ratio obtained from the Raman spectra of the samples [37], hence the defect content: As the ratio increases, the conductivity decreases (Figure 5b). The presence of structural defects can strongly influence the electronic properties of graphene like the charge carrier mobility, and therefore its electrical conductivity. Thus, previous works have reported a change in graphene behavior from metallic to semiconducting with increasing defect density [48]. The trend observed could also be explained considering that graphene defects alter the length of the interatomic bond. They also change the type of the hybrid trajectories of the partial carbon atoms, and these changes affect the electrical properties. Both point and single vacancy defects can act as electron scattering centers on the graphene surface, resulting in decreased conductivity [49].

### 3.3. Thermal Stability

**Figure 6:**
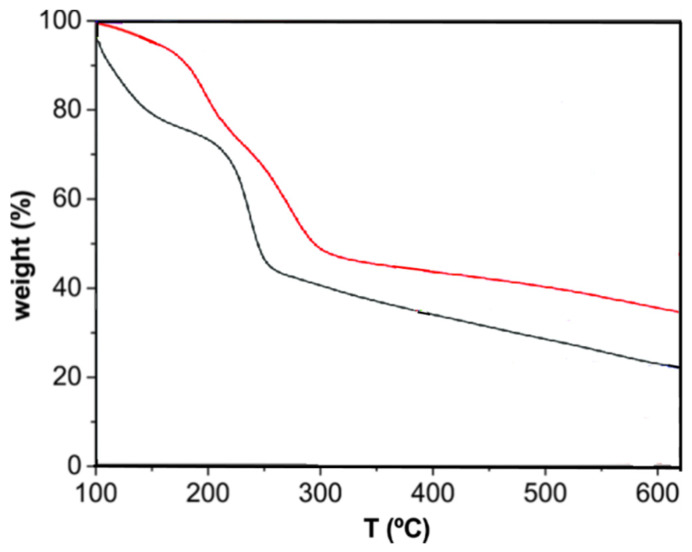
TGA thermograms under nitrogen for the reference GO * (black) and EGO 4.

TGA curves of the different EGOs were recorded under nitrogen and dry air atmospheres to obtain information about the influence of the synthesis conditions, hence the degree of oxidation, on the thermal stability of these materials, a property that is very important from an application viewpoint. Representative thermograms under nitrogen for the reference GO and EGO 4 are shown in Figure 6. GO starts to degrade (T_i_, taken at 2% weight loss) at about 100 °C, and experiences about 25% weight loss below 200 °C due to the evaporation of adsorbed water and the release of water molecules included within the GO structure [2]. Besides, about 30% mass loss is found between 200 and 250 °C, ascribed to the decomposition of oxygen-functional groups at the GO surface including epoxide, hydroxyl, and ketone groups on the basal planes and carboxyl moieties at the borders, as corroborated by the FT-IR spectrum [37]. On the other hand, the gradual mass loss above 250 °C can be ascribed to the removal of additional functional groups.

According to the FT-IR spectra [37], the EGOs have the same type of oxygenated functional groups as raw GO except for ether moieties that were detected in the samples with the highest level of oxidation. Thus, the shape of the thermograms for the EGOs was very similar to that of the reference GO, albeit shifted towards higher or lower temperatures. For instance, in the case of EGO 4, the curve is displaced to upper temperature. In particular, the initial degradation temperature T_i_ increased by about 30 °C compared to the GO synthesized by the Hummer’s method. Surprisingly, this EGO presents lower C/O ratio, that is, higher oxygen content than the reference GO, and it could be expected that the higher the number of oxygenated groups, the lower the thermal stability. Therefore, it seems that other factors besides the oxidation level influence the thermal stability. In fact, it has been reported that it should be distinguished between the decomposition of the GO surface groups and the cleavage of the subjacent carbon framework due to the formation of structural defects [50]. Therefore, experimental data suggest that the presence of defects such as vacancies may be the leading cause for the loss in thermal stability.

**Figure 7:**
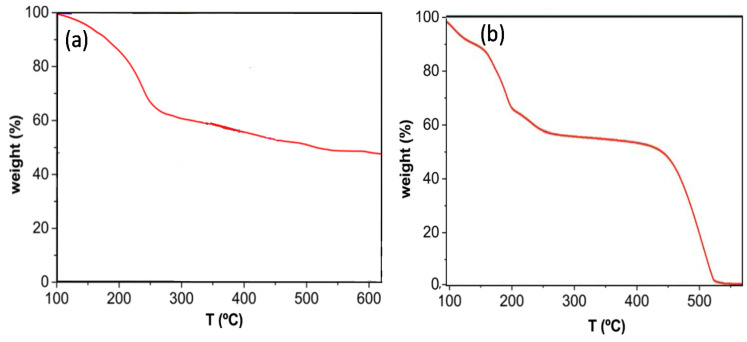
TGA thermograms under nitrogen (**a**) and air (**b**) for EGO 8.

TGA experiments were also performed under air conditions, as shown in Figure 7 for EGO 8. The characteristic degradation temperatures of all the synthesized EGOs are collected in Table 2. As can be observed, the thermal stability of GO is reduced in the presence of air, since the air accelerates the decomposition process. Thus, under air T_i_ decreases by an average of 30 °C, and the same trend is found for the maximum degradation temperature (T_max_) of each stage. Further, three weight losses can be detected, the third stage starts at around 450 °C and results in the total degradation of the material, in agreement with results reported by other authors [51]. During this stage, more than 50% of the material is removed, indicative of the high oxygen content.

**Figure 8:**
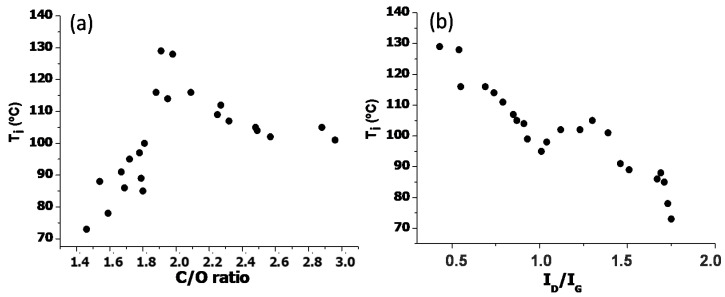
Initial degradation temperature (T_i_) obtained at 2 wt% weight loss versus the C/O ratio (**a**) and the I_D_/I_G_ band intensity ratio (**b**) for the synthesized EGOs.

To obtain more information about the effect of the level of oxidation and the surface defect content on the thermal stability, T_i_ was plotted against the C/O and I_D_/I_G_ ratio, as shown in Figure 8. Interestingly, this degradation temperature initially raises with increasing C/O ratio, showing a maximum value in the range of 1.9 to 2.1, and then decreases. It has been reported that the thermal stability depends on the arrangement of the oxygenated groups on the graphene surface (mainly on edges or defects) [52]. Thus, it could be that initially upon increasing the C/O ratio, the functional groups generated were located on the edges, thus hardly point defects would be introduced. These could be mainly carboxylic acids that have a strong tendency to form H-bonds, thereby stabilizing the G structure, and this could account for the small rise in T_i_. In fact, these samples have a low defect content. However, as the C/O ratio continues to rise, functional groups would be formed on point defects in the basal plane, within the hexagonal network, and this could result in reduced stability due to the disruption of the π conjugation.

On the other hand, the plot of T_i_ against the I_D_/I_G_ band intensity ratio shows a steady trend: The larger the number of defects, the lower the thermal stability of the sample. It has been reported that if a carbon atom on the graphene network becomes sp^3^ hybridized, assuming that it will be connected to three other carbon atoms and one hydrogen atom, the hydrogen defect generated will result in a noticeable drop in thermal conductivity (ca. 40% reduction in conductivity if the defect is introduced into 2.5% of the carbon atoms) [53]. Therefore, if the thermal conductivity is reduced, the heat dissipation would be significantly diminished as well, which at the end would be reflected in lower thermal stability. Overall, it seems that the thermal stability depends on both the degree of oxidation and the defect content, which should be tailored to attain a maximum stability. This stability enhancement is desirable for a wide number of applications ranging from batteries to sensors or thermal management in electronics systems.

### 3.4. Mechanical Properties

The extraordinary mechanical properties of graphene are one of the reasons that make it an ideal candidate as a reinforcing agent in composites [6]. In particular, its exceptional intrinsic mechanical properties, namely stiffness, strength, and toughness, lie in the stability of the sp^2^ bonds that form the hexagonal lattice and oppose a variety of in-plane deformations. Lee and coworkers [54] were pioneers in measuring the mechanical properties of free-standing monolayer graphene by nanoindentation in an AFM and recognized graphene as the strongest material ever measured, with a stiffness value close to 1 TPa and tensile strength of 130 GPa. In the case of GO, the functional groups tend to damage the graphene lattice and increase the interlayer thickness, which significantly reduces the intrinsic stiffness compared to pristine graphene. Thus, the effective Young’s modulus depends strongly on the functionalization degree, and values between 10 and 230 GPa have been reported [55]. Further, a strong dependence on the number of layers has been found, as well as on the applied strain.

**Figure 9:**
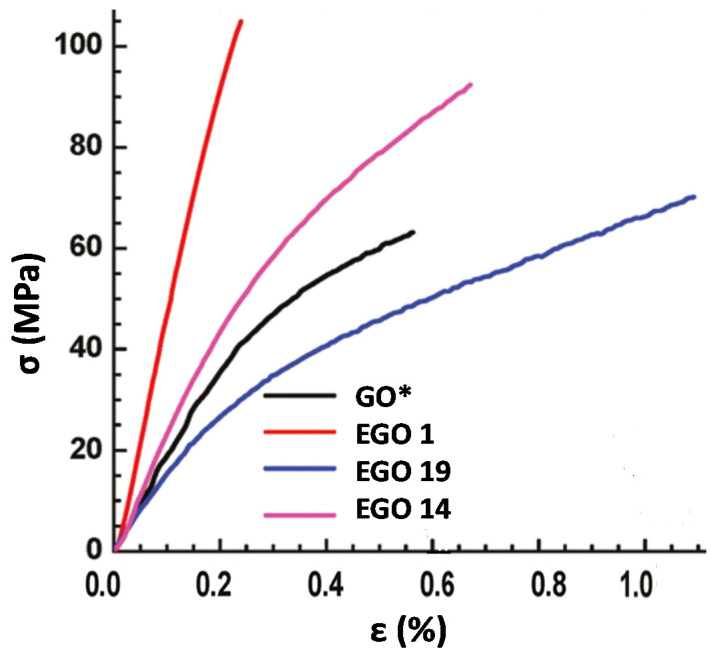
Typical stress–strain curves of the indicated samples.

To obtain more information about the influence of the defect content, oxidation level, and interlayer spacing on the macroscopic mechanical properties, namely the Young’s modulus and tensile strength, tensile tests were performed, and typical stress–strain curves of the reference GO, EGO 1, EGO 14, and 19 are displayed in Figure 9. Neat GO shows a region of elastic deformation and another of plastic deformation before the fracture. While defect-free graphene deforms purely elastically and exhibits a brittle failure, the cyclic epoxide groups on GO are believed to aid to dissipate strain energy and hamper crack propagation via a transformation from epoxide to ether group that takes place when GO sheets are subjected to an important mechanical stress, resulting in a ductile material with improved toughness [56]. This chemically induced plasticity in GO has been corroborated via density functional-based tight-binding (DFTB) calculations [57]. Nonetheless, higher epoxide percentage leads to an increase in the length of single and hybrid resonance bonds, resulting in reduced Young’s modulus [58]. This is consistent with our results, since EGO 15, with the highest percentage of epoxy groups according to the FT-IR spectra [37], shows one of the poorest mechanical properties.

Regarding the synthesized EGOs, different mechanical behaviors were found depending on the oxidation level and the defect content, as summarized in Table 3. For instance, EGO 1 with the lowest defect content and a moderate level of oxidation exhibited a clear brittle failure (Figure 9), with the highest Young’s modulus (ca. 55 GPa), hence material stiffness, and the highest tensile strength (ca. 102 MPa). Conversely, EGO 19, with very high defect content shows a more ductile behavior than the reference GO, and one of the lowest Young’s modulus (ca. 13 GPa), albeit has slightly higher tensile strength than the GO synthesized by the Hummer’s method. On the other hand, EGO 14, with a high level of oxidation though moderate defect content (lower than that of neat GO), displays an intermediate behavior.

Thus, it appears that not only the level of oxidation, but also the defect content, plays an important role on the mechanical properties of GO-based materials. In fact, albeit the stiffness in general decreases with decreasing the C/O ratio (increasing oxidation level), no direct correlation was found between them (Table 3). The same applied for the interlayer distance and the SSA. It could be expected that better exfoliated GO sheets, with higher SSA resulted in more intense interlocking between layers, hence better mechanical properties. However, no direct relationship was found between SSA and the Young’s modulus or strength (Table 3).

**Figure 10:**
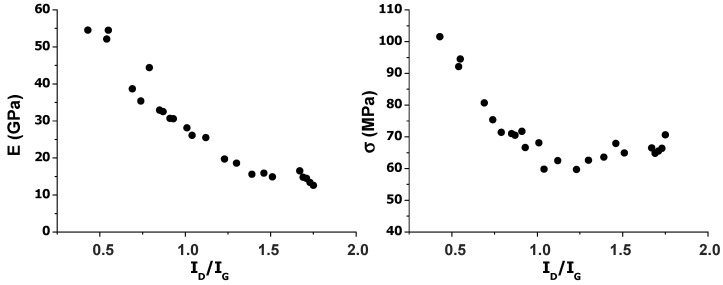
Young’s modulus (E) and tensile strength (σ) versus the I_D_/I_G_ band intensity ratio.

In contrast, a clear and steady drop in stiffness was found with increasing I_D_/I_G_ ratio, hence defect content, as shown in Figure 10. This corroborates that the material rigidity is strongly influenced by the surface defects. A similar trend was observed for the tensile strength, albeit in this case, the drop was more pronounced at low defect contents, while the properties hardly changed at high defect density. This is consistent with previous studies that investigated the correlation between graphene surface area, presence of functional groups, and defect content on the performance of nanocomposites [59]. Thus, it was reported that dangling bonds in single vacancy and double vacancy defected graphene, that is, the presence of immobilized free radicals, strongly decreased its mechanical properties, especially the stiffness and fracture strain. It was found that Young’s modulus with defects was about 43% lower than that of non-defective graphene. The presence of defects, particularly vacancies, causes the bending of the graphene sheet, resulting in reduced stiffness. Further, mechanical failure is typically dictated by the more defective regions. This behavior is consistent with the results observed in this work: Upon increasing the number of defects in the G basal plane, the stiffness drops drastically. It is expected that upon functionalization of the graphene lattice with carbonyl, epoxide, and ether groups, rupture of some C–C bonds would take place, which would introduce discontinuities in the graphene lattice, thus lowering the material rigidity. However, the strength, that is, the maximum amount of stress the material can take before failure, remains merely unchanged for I_D_/I_G_ ratios higher than 1, indicative that this property is less sensitive to the defect content. According to the literature [60], I_D_/I_G_ ratios lower than 1 correspond to a sp^3^-defect regime, with sp^3^ point defects in the form of oxygenated functional groups, while ratios higher than 1 are ascribed to a vacancy-defect regime with many nanocavities or nanopores. Our experimental results suggest that these nanocavities have more influence on the stiffness than on the strength of the nanomaterial. Overall, it is clear that the defect density strongly influences the mechanical properties of these nanomaterials.

## 4. Conclusions

EGOs with various amounts of oxygenated groups have been synthesized by an electrochemical two-step process: Intercalation followed by oxidation/exfoliation. For comparative purposes, a reference GO was prepared via a modified Hummers’ method. The resulting GO samples have been characterized by different techniques in order to correlate their surface morphology with the macroscopic properties. SEM and AFM images reveal that the synthesis procedure used herein preserves the integrity of the graphene sheets, allowing to obtain large, homogenous, and exfoliated GO layers. The macroscopic properties have been found to depend on the surface topography, interlayer spacing, defect content, and specific surface area. The electrical resistance decreases with increasing specific surface area although rises with increasing the D/G band intensity ratio obtained from the Raman spectra, hence the defect content. The thermal stability of the EGOs under inert and oxidative environments depends on the C/O ratio and the defect content: The larger the number of defects, the lower the thermal stability of the nanomaterial. Their macroscopic mechanical properties measured via stress–strain curves are also strongly influenced by the defect content. The Young’s modulus and tensile strength drop significantly with increasing the D/G band intensity ratio, while no direct correlation was found with their specific surface area, level of oxidation, or interlayer spacing. Young moduli values as high as 54 GPa have been measured, which corroborates that the developed method preserves the integrity of the graphene flakes. The approach developed herein provides an effective mean to tailor the physical properties of nanomaterials incorporating GO for a wide variety of applications.

## Figures and Tables

**Table 1:** Nomenclature and experimental conditions for the different exfoliated graphene oxide (EGO) samples.

Sample	Voltage I/II (V)	Time I/II (min)	H_2_SO_4_ (wt%)	C/O Ratio	*d*_001_ (nm)	Sample	Voltage I/II (V)	Time I/II (min)	H_2_SO_4_ (wt%)	C/O Ratio	*d*_001_ (nm)
GO *	-	-	-	2.25	0.8615	EGO 12	2.0/20	10/0.5	98	1.59	0.9218
EGO 1	1.0/10	10/1	65	2.09	0.8816	EGO 13	2.0/20	10/1	40	1.80	0.9378
EGO 2	1.0/10	10/2	65	1.98	0.8956	EGO 14	2.0/20	10/1	65	1.67	0.9595
EGO 3	1.0/20	10/0.5	98	1.72	0.9034	EGO 15	2.0/20	10/1	98	1.46	0.9230
EGO 4	1.0/20	10/1	40	1.91	0.9135	EGO 16	2.0/20	10/2	40	1.78	0.9167
EGO 5	1.0/20	10/1	65	1.79	0.9145	EGO 17	2.0/20	10/2	65	1.69	0.9496
EGO 6	1.0/20	10/1	98	1.54	0.9187	EGO 18	2.0/30	10/0.5	65	2.49	0.8583
EGO 7	1.0/30	30/1	40	2.32	0.8594	EGO 19	2.0/10	30/0.5	98	2.48	0.8472
EGO 8	1.0/30	30/2	65	2.57	0.8576	EGO 20	2.0/20	30/2	65	2.27	0.8602
EGO 9	2.0/10	10/1	65	1.88	0.9161	EGO 21	2.0/30	30/1	40	2.88	0.8564
EGO 10	2.0/10	10/2	65	1.81	0.9356	EGO 22	2.0/30	30/2	65	2.96	0.8499
EGO 11	2.0/20	10/0.5	65	1.95	0.9021						

* Synthesized by a modified Hummers’ method. I and II refer to the intercalation and exfoliation stages, respectively. The C/O ratio has been calculated from elemental analysis measurements, and *d* is the interlayer spacing corresponding to the (001) reflection of GO.

**Table 2:** Characteristic degradation temperatures of the EGOs obtained from TGA experiments under nitrogen and dry air atmospheres.

Sample	T_i_ (°C) N_2_	T_max_ (°C) N_2_	T_i_ (°C) O_2_	T_max_ (°C) O_2_	C/O Ratio	Sample	T_i_ (°C) N_2_	T_max_ (°C) N_2_	T_i_ (°C) O_2_	T_max_ (°C) O_2_	C/O Ratio
GO *	97	240	75	210	2.25	EGO 12	78	219	59	186	1.59
EGO 1	116	263	96	237	2.09	EGO 13	85	220	66	190	1.80
EGO 2	128	276	102	244	1.98	EGO 14	91	234	70	203	1.67
EGO 3	95	236	72	209	1.72	EGO 15	73	209	55	191	1.46
EGO 4	129	271	99	243	1.91	EGO 16	97	238	76	76	1.78
EGO 5	89	233	66	205	1.79	EGO 17	86	232	67	198	1.69
EGO 6	88	229	68	200	1.54	EGO 18	104	245	85	215	2.49
EGO 7	107	249	88	220	2.32	EGO 19	105	246	84	219	2.48
EGO 8	116	262	93	236	2.57	EGO 20	112	257	93	234	2.27
EGO 9	116	263	95	233	1.88	EGO 21	105	247	86	218	2.88
EGO 10	100	241	85	212	1.81	EGO 22	101	242	83	214	2.96
EGO 11	114	260	65	229	1.95						

Ti: Initial degradation temperature at 2 wt% weight loss. T_max_: Temperature of maximum rate of weight loss. The C/O ratio has been calculated from elemental analysis measurements.

**Table 3:** Mechanical properties of the EGOs obtained from tensile experiments and their relation to the specific surface area (SSA), interlayer distance *d*, and C/O ratio.

Sample	E (GPa)	σ (MPa)	SSA (nm)	*d*_001_ (nm)	C/O Ratio	Sample	E (GPa)	σ (MPa)	SSA (nm)	*d*_001_ (nm)	C/O Ratio
GO *	26.1	60.2	12.3	0.8615	2.25	EGO 12	13.4	66.3	23.1	0.9218	1.59
EGO 1	54.5	102	11.2	0.8816	2.09	EGO 13	39.3	70.5	26.5	0.9378	1.80
EGO 2	52.1	95.6	13.2	0.8956	1.98	EGO 14	32.9	90.9	32.9	0.9595	1.67
EGO 3	28.1	61.5	15.3	0.9034	1.72	EGO 15	13.6	65.4	24.0	0.9230	1.46
EGO 4	51.1	93.2	18.6	0.9135	1.91	EGO 16	30.6	67.8	20.4	0.9167	1.78
EGO 5	42.9	75.6	19.1	0.9145	1.79	EGO 17	29.5	61.4	28.7	0.9496	1.69
EGO 6	14.8	65.7	16.3	0.9187	1.54	EGO 18	20.7	68.7	7.1	0.8583	2.49
EGO 7	22.3	63.4	8.6	0.8594	2.32	EGO 19	12.5	70.1	5.1	0.8472	2.48
EGO 8	26.8	68.7	7.9	0.8576	2.57	EGO 20	19.7	64.3	9.78	0.8602	2.27
EGO 9	38.7	70.2	20.1	0.9161	1.88	EGO 21	18.6	63.8	6.7	0.8564	2.88
EGO 10	44.4	80.2	24.7	0.9356	1.81	EGO 22	15.6	62.5	5.8	0.8499	2.96
EGO 11	35.3	70.1	19.9	0.9021	1.95						

E: Young’s modulus; σ: Tensile strength; *d*: Interlayer spacing.
